# Unveiling the potential impact of RNA m5C methyltransferases NSUN2 and NSUN6 on cellular aging

**DOI:** 10.3389/fgene.2025.1477542

**Published:** 2025-04-16

**Authors:** Jiale Zhou, Yang Han, Fuxi Ji, Renquan Zhang, Yuru Liang, Xudong Zhao, Ruizhe Hou

**Affiliations:** ^1^ Department of Neurosurgery, China-Japan Union Hospital of Jilin University, Changchun, China; ^2^ Key Laboratory of Zoonosis Research, Ministry of Education, College of Veterinary Medicine, Jilin University, Changchun, China; ^3^ Laboratory Animal Center, College of Animal Science, Jilin University, Changchun, China; ^4^ Department of Critical Care, The Second Hospital of Jilin University, Changchun, China

**Keywords:** RNA methylation, 5-methylcytosine, NSun2, NSUN6, aging

## Abstract

NSUN2 and NSUN6, two family members of NOL1/NSUN protein, are mainly responsible for catalyzing the formation of 5-methylcytosine (m5C) in RNA and highly involved in the physiological and pathological processes of many diseases. To investigate the biological functions of NSUN2 and NSUN6, NSUN2^−/−^ and NSUN6^−/−^ HEK293T cell lines were separately constructed by CRISPR/Cas9. We found no significant interaction between the protein expression of NSUN2 and NSUN6. Notably, the ablation of NSUN2 or NSUN6 reduced cell proliferation and increased expression of the senescence-associated marker P27, whereas more β-galactosidase-positive cells were observed in response to H_2_O_2_-induced oxidative stress. Moreover, the expression of NSUN2 and NSUN6 was significantly reduced in the HGPS premature aging cell lines by the LMNA^G609G^ mutation. Taken together, we demonstrated that NSUN2 and NSUN6 may be inextricably linked to cellular aging and thus provide potential novel strategies for the clinical therapeutics of aging and age-associated disease in the future.

## 1 Introduction

The RNA m5C methylation modification, which adds a donor active methyl group to the 5th carbon of a cytosine base in RNA, has been considered one of the most important RNA modifications since its discovery more than four decades ago (Dubin and Taylor, 1975; Desrosiers et al., 1974). As integral components of the RNA m5C methylation modification regulatory network, methyltransferases (“writers”), demethylases (“erasers”), and m5C-binding proteins (“readers”) dynamically regulate m5C modifications. Numerous studies have revealed multiple molecular functions of m5C in many key RNA metabolic stages such as mRNA stabilization, translation and nuclear export (Yang et al., 2017; Yang et al., 2019; García-Vílchez et al., 2019; Trixl and Lusser, 2019; Boo and Kim, 2020; Courtney et al., 2019). Moreover, the dynamics of m5C plays an integral role in many physiological and pathological processes such as early embryonic development (Liu et al., 2022), neurodevelopmental disorders (Chen P. et al., 2019; Flores et al., 2017) and the genesis and migration of various tumors (Chen et al., 2022; Chen X. et al., 2019; Yang et al., 2021; Li et al., 2023).

NSUN2 and NSUN6 are two major methyltransferases in RNA responsible for catalyzing the methylation of m5C. NSUN2, as the most widely studied methyltransferase in the NSUN family of proteins, is mainly located in the nucleus, has abundant targets, and plays an important role in a variety of biological processes, such as cell proliferation, the stress response, metabolism, cell migration, and differentiation (Flores et al., 2017; Xing et al., 2015; Gkatza et al., 2019; Blanco et al., 2014). Previous studies have shown that NSUN6 is primarily responsible for catalyzing the formation of m5C at position C72 in tRNA^Cys^ and tRNA^Thr^ (Haag et al., 2015). Subsequently, with the development of sequencing technology, NSUN6 was discovered to also be a m5C methyltransferase with strong substrate specificity for mRNAs, generally with the motif feature of “CTCCA” (Liu et al., 2022; Selmi et al., 2021).

Aging, the gradual decline in an organism’s physiological functions, is an inevitable progression toward natural death and holds significant biological importance (Li et al., 2022). As the population ages, it has emerged as a prominent health concern. Numerous diseases including cancer, diabetes, cardiovascular diseases and neurodegenerative diseases, are closely linked to the aging process. Consequently, investigating key regulators influencing aging is imperative for comprehending its mechanisms, enhancing human health, and managing aging-related diseases. Although previous studies have extensively explored the epigenetic connections between DNA methylation and aging, only a few studies focused on the association between RNA methylation, its associated proteins, and aging (Li et al., 2022; Noroozi et al., 2021). Thus, further investigation into the regulatory mechanisms of RNA methylation and its related proteins in aging-related diseases holds promise for unveiling novel approaches to combat aging.

In this study, NSUN2^−/−^ and NSUN6^−/−^ HEK293T cell lines were successfully constructed to explore the biological functions of NSUN2 and NSUN6. The deletion of NSUN2 and NSUN6 led to reduced cell proliferation and the appearance of senescence-associated phenotypes. Additionally, the expression levels of NSUN2 and NSUN6 were significantly decreased in a cellular model of Hutchinson-Gilford Progeria Syndrome (HGPS). These findings revealed that NSUN2 and NSUN6 may be implicated in the regulation of cellular senescence. Future in-depth investigation into the regulatory mechanisms of NSUN2 and NSUN6 on aging may be essential for developing treatments for aging-related diseases.

## 2 Materials and methods

### 2.1 Cell culture and plasmid transfection

HEK293T cells (ATCC) and Neuro-2a (ATCC) cells were cultured in Dulbecco’s Modified Eagle Medium (DMEM) supplemented with 10% fetal bovine serum (CLARK BIOSCIENCE) and 1% penicillin (100 U/mL) and streptomycin (100 μg/mL). The cells were seeded in 6-well plates and transfected using Hieff Trans™ Liposomal Transfection Reagent (Yeasen).

NSUN2-depleted cell lines were generated by cloning NSUN2-targeting single guide RNA sequences into the PX459 plasmid. Plasmids were then transfected into HEK293T cells and puromycin (Meilunbio) was added at a final concentration of 3 μg/mL to enrich the positively transfected cells 24 h after transfection. After 72 h, the cells were collected and used for genotyping by Sanger sequencing. NSUN6-depleted cell lines were generated in the same way. The LMNA^G609G^ cell line was constructed by co-transfecting BE3 with the corresponding sgRNA to introduce a point mutation. Monoclonal cell screening and characterization were then performed to identify the LMNA^G609G^ cell line. Primers and single guide RNA sequences used for genotyping are listed in Supplementary Table S1.

### 2.2 Cell viability measurements

Cells were seeded at 1,000 cells per well in 96-well plates. Following cell attachment to the well and subsequent normal growth, cell viability was assessed by the Cell Counting Kit-8 (CCK-8; Meilun Bio, China) and MTT assay kit (Meilun Bio, China) according to the manufacturers’ protocols. The CCK8 kit assay analyzes cellular activity by measuring its absorbance at 450 nm. The MTT kit determines its light absorbance value at 562 nm using an enzyme marker.

$$\text{cell viability }\left(\%\right)=\left[\left(\text{As}-\text{Ab}\right)/\left(\text{Ac}-\text{Ab}\right)\right]\times 100$$

(As = Absorbance of experimental wells, Ab = Absorbance of blank wells, Ac = Absorbance of control wells).

### 2.3 Western blotting

For protein blotting, samples were lysed with RIPA lysis buffer (Meilun Bio) spiked with phenylmethylsulfonyl fluoride (PMSF) and repeatedly injected and pumped with 1 mL syringe. Total protein extracts were separated by SDS-PAGE on a 12% gel and then transferred to 0.45 nm polyvinylidene fluoride membranes (Boster). Subsequently, the proteins were probed with specific antibodies after the blot was blocked with 5% non-fat milk (Boster). Images were quantified using ImageJ software and all data are expressed as mean ± SEM.

The following antibodies and concentrations were used: NSUN2 Polyclonal antibody (Proteintech; Cat No.20854-1-AP; 1:7500), NSUN6 Polyclonal antibody (Proteintech; Cat No. 17240-1-AP; 1:2000), Rabbit Anti-GAPDH antibody (Bioss; bs-41373R; 1:2000), P27 Polyclonal antibody (Proteintech; Cat No. 25614-1-AP; 1:2000), HRP-labeled Goat Anti-Rabbit IgG (H + L) (Beyotime Biotechnology; A0208; 1:10000).

### 2.4 GO and KEGG analysis

GO and KEGG analyses of mRNAs with m5C modifications were performed using the DAVID bioinformatics database. P-values less than 0.05 were considered statistically significant (Sherman et al., 2022).

### 2.5 SA-β-galactosidase activity

WT, NSUN2^−/−^ and NSUN6^−/−^ HEK293T cells were exposed to H_2_O_2_ (150 μM) for 4 h and subjected to SA-β-gal analysis. SA-β-galactosidase activity (β-gal staining) was carried out according to the instructions of the kit (Solarbio).

### 2.6 Statistical analysis

All data are expressed as mean ± SEM of three independent determinations. Data were analyzed through a two-tailed t-test. A probability of *p* < 0.05 was considered statistically significant; *, *p* < 0.05; **, *p* < 0.01; ***, *p* < 0.001; and ****, *p* < 0.0001 denote the significance thresholds; ns denotes not significant.

## 3 Results

### 3.1 Establishment of NSUN2^−/−^ and NSUN6^−/−^ HEK293T cell lines

**FIGURE 1:**
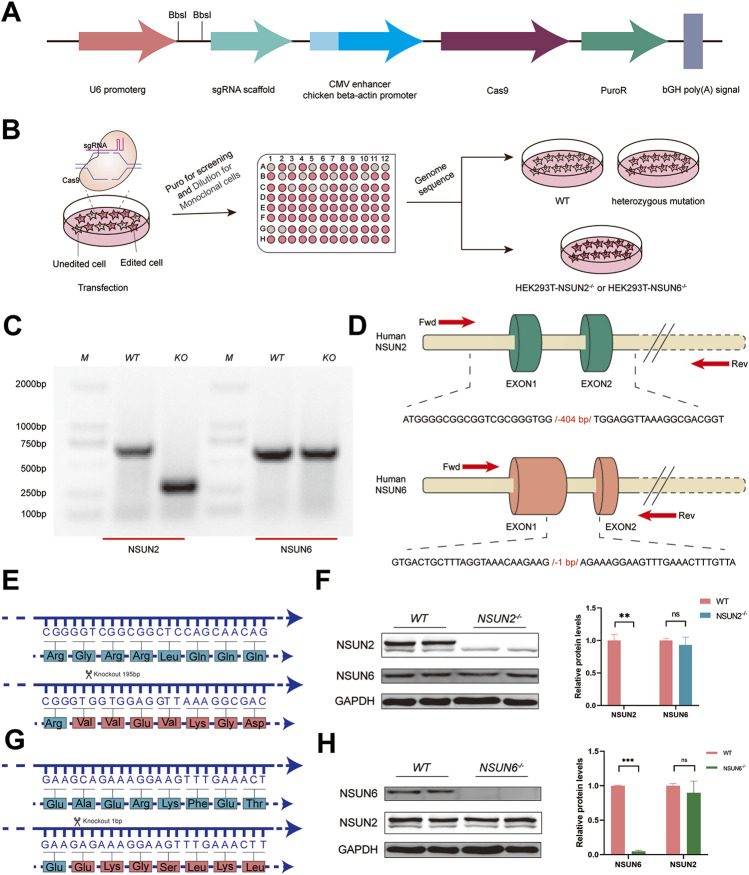
CRISPR/Cas9 system-mediated NSUN2 and NSUN6 depleted in HEK293T. **(A)** Schematic diagram of plasmid construction of PX459 for knockdown of NSUN2 and NSUN6 genes using CRISPR/Cas9 technology. **(B)** Schematic representation the construction of NSUN2^−/−^ and NSUN6^−/−^ Cell line. **(C)** Agarose gel electrophoresis results of PCR products to verify the knockdown of NSUN2 and NSUN6. M represents the DNA Marker DL2000 used. **(D)** Schematic representation of knockout targeting the human methyltransferase NSUN2 and NSUN6 genes. NSUN2 and NSUN6 exons are indicated by columns, primers F and R were used to assay knockout efficiency, and the sequence information below shows the number of missing bases. **(E, G)** Schematic representation of the amino acids encoded by the wild-type, NSUN2^−/−^ **(E)** and NSUN6^−/−^ **(G)** HEK293T cell lines, with changes in the encoded synthesized proteins after base deletions. **(F, H)** The knockout efficiency of NSUN2 **(F)** and NSUN6 **(H)** in HEK293T cell lines verified by Western blotting. The protein level of GAPDH was served as loading controls.

NSUN2 (Zhang et al., 2021) and NSUN6 (Liu et al., 2021), two family members of the NOL1/NSUN protein, were both identified as m^5^C methyltransferases of mRNA (Schumann et al., 2020). To investigate the biological functions of NSUN2 and NSUN6, NSUN2^−/−^ and NSUN6^−/−^ HEK293T cell lines were obtained using the PX459 system (Figure 1A). Plasmids were transfected into HEK293T cells for knockout assays using the PX459 system, and then cells were diluted and seeded into 96-well plates for monoclonal screening using the doubling dilution method (Figure 1B). The monoclonal cells were then selected and genome sequencing was performed. PCR results showed that 404bp was missing in the NSUN2^−/−^ HEK293T cell line, and 1bp was missing in the NSUN6^−/−^ HEK293T cell line (Figures 1C, D). Following the successful construction of NSUN2^−/−^ and NSUN6^−/−^ HEK293T cell lines, we examined the expression of NSUN2 and NSUN6 at the protein level. Analysis of the knockout sequences revealed that the base deletions led to a frameshift, preventing proper translation of NSUN2 and NSUN6 proteins (Figures 1E, G). Western blot (WB) analysis confirmed the absence of NSUN2 expression in the NSUN2^−/−^ HEK293T cell line, without affecting NSUN6 expression (Figure 1F). Similarly, NSUN2 expression remained comparable to that in wild-type cells when NSUN6 expression was absent in the NSUN6^−/−^ HEK293T cell line (Figure 1H). These suggest that although both NSUN2 and NSUN6 methyltransferases catalyze the m5C modification, their protein expression levels do not appear to interact significantly.

### 3.2 Functional analysis of NSUN2 and NSUN6

**FIGURE 2:**
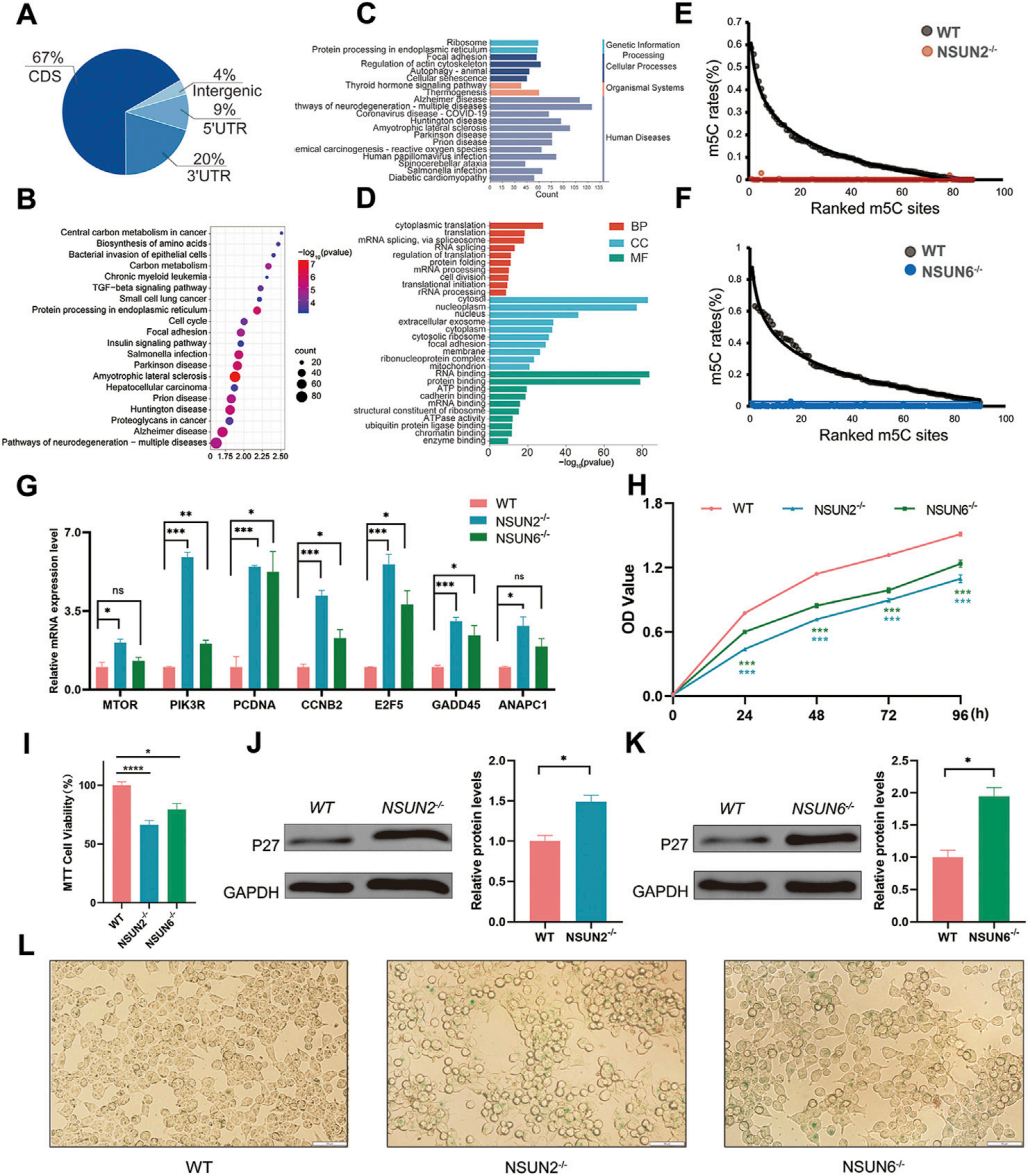
NSUN2 and NSUN6 deletions may have an effect on cellular senescence. **(A)** Pie chart showing the distribution of m5C sites in different transcript regions. **(B, C)** Genes with RNA m5C modifications were analysed for KEGG bioprocess enrichment. Statistical analyses were performed using the DAVID tool. **(D)** Genes with RNA m5C modifications were analysed for Gene Ontology (GO) biological processes, cellular components, and molecular functional enrichment. Statistical analyses were performed using the DAVID tool. **(E)** m5C methylation levels at sites measured from wild type and NSUN2^−/−^ cells. **(F)** m5C methylation levels at sites measured from wild type and NSUN6^−/−^ cells. **(G)** RT-qPCR analysis of relative mRNA expression levels of MTOR, PIK3R, PCDNA, CCNB2, E2F5, GADD45, and ANAPC1 in wild-type (WT), NSUN2^−/−^, and NSUN6^−/−^ HEK293T cell lines. **(H)** Cell proliferation was determined by CCK8 assay in WT, NSUN2^−/−^ and NSUN6^−/−^ HEK293T cell lines. **(I)** The viability cells were evaluated using the MTT Cell Proliferation Assay. **(J, K)** Expression of P27 in NSUN2^−/−^ and NSUN6^−/−^ HEK293T cell lines was verified by Western blotting. GAPDH protein levels were used as a loading control. **(L)** WT, NSUN2^−/−^ and NSUN6^−/−^ HEK293T cells were exposed to H_2_O_2_ (150 μM) for 4 h and subjected to SA-β-gal analysis.

As methyltransferases of RNA m5C, the biological roles of NSUN2 and NSUN6 are intricately linked to the function of genes bearing RNA m5C methylation modifications. To further understand these roles, we then integrated data from three transcriptome-wide RNA m5C methylation studies conducted on the HEK293T cell line (Dai et al., 2024; Huang et al., 2019; Sun et al., 2019). Our analysis revealed that the m5C loci were predominantly enriched in the coding sequence (CDS) region (67%) (Figure 2A). Additionally, 20% of the m5C loci were located in the 3′untranslated region (UTR), with a minimal presence in the 5′UTR and intergenic regions (Figure 2A).

Gene Ontology (GO) and Kyoto Encyclopedia of Genes and Genomes (KEGG) analysis were carried out on genes with m5C modifications, indicating their involvement in key biological processes such as cell division, cell cycle regulation, mRNA splicing, and translation (Figures 2B–D). Notably, genes with m5C modifications were significantly enriched in pathways related to cell cycle regulation, senescence, and senescence-associated diseases. These modifications appear to have a crucial impact on cell cycle regulation and senescence. To determine the role of NSUN2 and NSUN6 in the regulation of m5C modification, we performed a comprehensive analysis of m5C methylation levels at predicted sites in wild-type, NSUN2^−/−^, and NSUN6^−/−^ HEK293T cell lines, using data from an independent study on m5C methylation sequencing in HEK293T cells (Liu et al., 2021). The analyses indicate that the deletion of NSUN2 or NSUN6 leads to a near-complete loss of methylation at the predicted m5C sites (Figures 2E, F). This further confirms that NSUN2 and NSUN6 are the primary methyltransferases responsible for mRNA methylation and highlights the significance of investigating their biological functions.

Cell proliferation and cycling are essential parameters for assessing cellular metabolism and physiopathological functions. To determine whether NSUN2 and NSUN6 influence cell cycle regulation and senescence, we performed RT-qPCR analyses of key genes in pathways associated with cell cycle regulation and cellular senescence. These pathways were significantly enriched for m5C-modified genes. The results revealed that the mRNA levels of these key genes were mostly altered in the methyltransferase-deficient cells compared to the wild type (Figure 2G). Subsequently, we assessed cell proliferation using the CCK8 assay. The results showed a significant reduction in cell proliferation following the deletion of NSUN2 and NSUN6 compared to wild-type cells (Figure 2H). Similarly, the MTT assay demonstrated a marked decrease in cell viability in NSUN2^−/−^ and NSUN6^−/−^ HEK293T cell lines (Figure 2I). Furthermore, examination of senescence markers revealed that the absence of these methyltransferases significantly increased the expression of the senescence-associated marker P27 (Figures 2J, K). Additionally, knockdown of NSUN2 or NSUN6 significantly elevated the senescence-associated β-galactosidase activity after H_2_O_2_ treatment (Figure 2L). In conclusion, our results suggest that deficiency in NSUN2 and NSUN6 methyltransferase leads to the development of senescence-associated phenotypes.

### 3.3 NSUN2 and NSUN6 may play an important role in the regulation of aging

**FIGURE 3:**
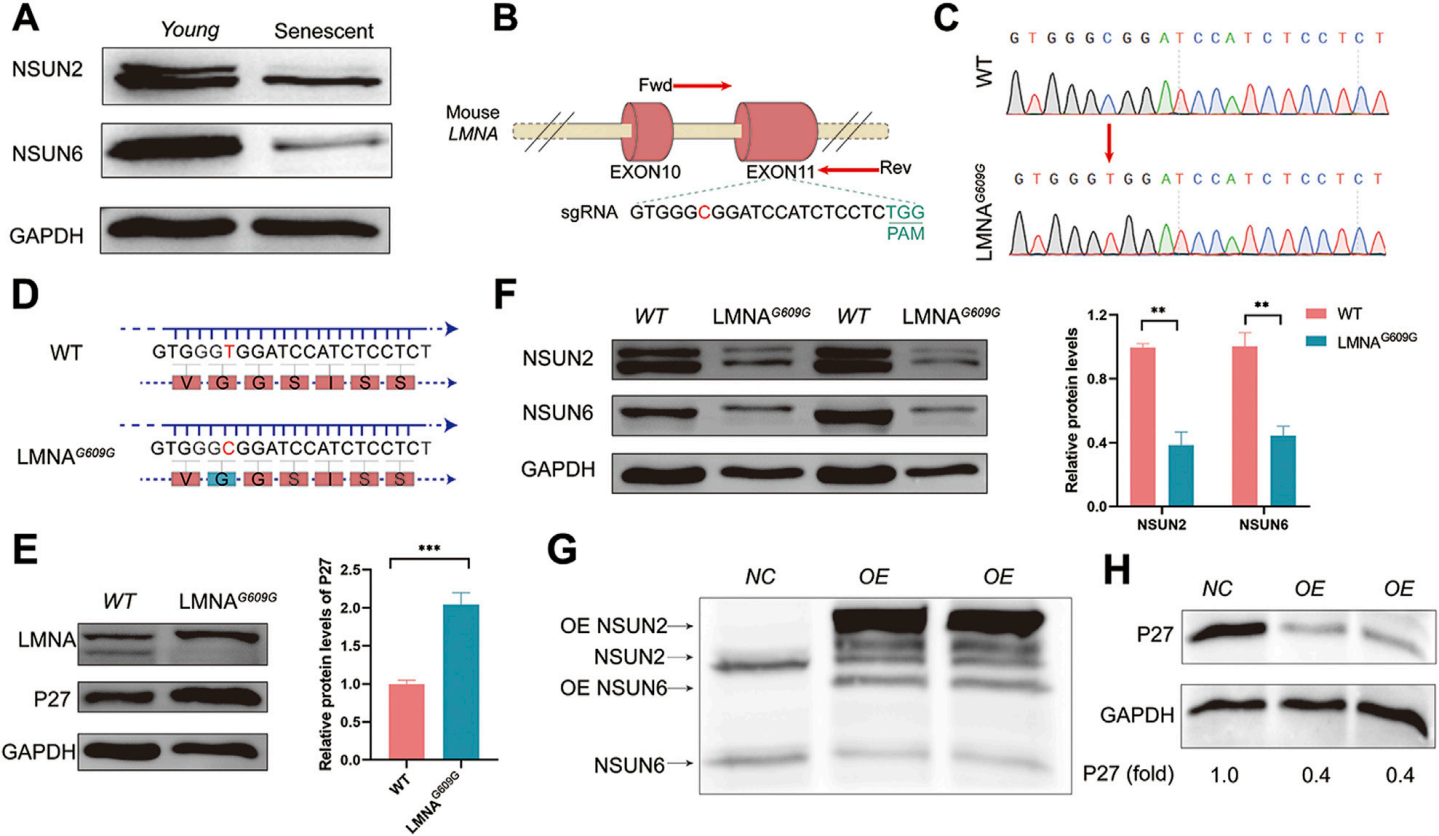
Potential role of NSUN2 and NSUN6 in the regulation of senescence. **(A)** Protein levels of NSUN2, NSUN6 and GAPDH were analyzed by Western blotting in early-passage (Young) and late-passage (Senescent) MEF cells. **(B)** Schematic representation of the G609G mutation generated against the Neuro-2a cell line LMNA gene. Primers F and R were used to detect the mutation efficiency of the gene and the sequence information below shows the number of missing bases. Sequences labelled in black below are sgRNA sequences. **(C)** Sanger sequencing results of two genotypes. **(D)** Schematic representation of amino acids encoded by wild type and LMNA^G609G^ Neuro-2a cell line. **(E, F)** WB shows the relative level of the compared protein level of LMNA, P27, NSUN2 and NSUN6 among wild-type and LMNA^G609G^ Neuro-2a cell lines. GAPDH protein levels were used as a loading control. The corresponding quantitative results are shown on the right, and the data are the mean ± S.E.M of three independent experiments. **(G, H)** LMNA^G609G^ Neuro-2a cells were co-transfected with pCMV-NSun2 vector and pCMV-NSun6 vector. Cell lysates were prepared and analysed by Western blotting to assess the levels of NSUN2 **(G)**, NSUN6 **(G)**, P27 **(H)** and GAPDH **(H)** proteins. Untreated LMNA^G609G^ cells were labelled as NC group.

To investigate whether NSUN2 and NSUN6 play roles in senescence regulation, we assessed the levels of NSUN2, NSUN6 and GAPDH proteins in early-passage (Young) and late-passage (Senescent) MEF cells by Western blot analysis. As shown in Figure 3A, the levels of NSUN2 and NSUN6 decreased during replicative senescence.

We next examined the protein levels of NSUN2 and NSUN6 in another aging model. A point mutation (G609G) in the mouse LMNA gene leads to a mis-splicing event characteristic of Hutchinson–Gilford progeria syndrome (HGPS) (Zhao et al., 2023). LMNA^G609G^ serves as an excellent model for studying HGPS. To further investigate, we designed single-guide RNAs (sgRNAs) targeting exon 11 of the LMNA gene (Figure 3B) and generated a Neuro-2a cell line expressing LMNA^G609G^ (Figures 3C, D). Verification at the protein level confirmed that the LMNA^G609G^ Neuro-2a cell line, similar to previous studies, exhibited a deficiency in LMNA protein expression and displayed a senescence phenotype, marked by a significant increase in the expression of the senescence-associated marker P27 (Figure 3E). Particularly, in the HGPS model, we observed a pronounced decrease in the protein expression levels of both NSUN2 and NSUN6 (Figure 3F). To investigate whether the decreased expression levels of NSUN2 and NSUN6 are a consequence of senescence or if they play a role in senescence regulation, we successfully transiently overexpressed NSUN2 and NSUN6 in LMNA^G609G^ Neuro-2a cells (Figure 3G). Notably, the protein levels of P27 significantly decreased with the increase in NSUN2 and NSUN6 expression (Figure 3H). This further supports the notion that NSUN2 and NSUN6 may play crucial roles in the regulation of aging.

## 4 Discussion

In recent years, RNA m5C methylation modification has gained increasing attention as a significant post-transcriptional modification closely linked to the pathogenesis of various diseases (Zhang et al., 2021). NSUN2 and NSUN6 are the primary m5C methyltransferases responsible for catalyzing m5C formation in mRNA. Numerous studies have explored the biological functions of NSUN2 and NSUN6 by reducing their expression levels (Tang et al., 2015; Zhang et al., 2012). However, this approach has limitations, as it may not fully elucidate the role of these methyltransferases in the regulation of related diseases, potentially overlooking diseases with low association. Furthermore, few studies have simultaneously focused on both NSUN2 and NSUN6, resulting in a somewhat one-sided understanding of their functions.

Although individual studies have demonstrated that NSUN2 regulates cellular senescence, the function of other RNA m5C methyltransferases in this process has not been clearly demonstrated. Therefore, our study comprehensively confirmed the association between RNA m5C methyltransferases and cellular senescence by individually knocking down the two most common RNA m5C methyltransferases, NSUN2 and NSUN6. Functional analysis of methyltransferase-deficient cells revealed that deletion of NSUN2 and NSUN6 led to reduced cell proliferation, increased expression of senescence-associated marker P27, and a higher number of β-galactosidase-positively cells following H_2_O_2_-induced oxidative stress. Furthermore, we observe a significant decrease in NSUN2 and NSUN6 expression in HGPS senescent cells caused by the LMNA^G609G^ mutation. This highlights the potential key role of RNA m5C methylation in senescence. However, our study did not conclusively establish whether the regulation of senescence by NSUN2 and NSUN6 is directly attributed to methylation. Further experiments are needed to verify whether the regulation of aging by these two NSUN proteins is mediated by methylation or alternative pathways.

Nevertheless, this study only provides preliminary insights into the relationship between NSUN protein and cellular aging. Although previous studies have shown that NSUN2 can delay replicative senescence by inhibiting P27 translation (Tang et al., 2015), the mechanism by which NSUN6 affects senescence have not been detailed. Therefore, it appears to be necessary to investigate the specific mechanisms by which NSUN6 regulates senescence. In addition, future research would also confirm whether NSUN2 and NSUN6 synergistically regulate aging, albeit this study demonstrates that protein expression levels of these two enzymes do not significantly affect each other. Understanding the combined effects of these two RNA m5C methyltransferases on the aging process could provide deeper insights into the molecular mechanisms of aging and reveal new therapeutic targets for aging-related diseases.

## 5 Conclusion

In this study, we successfully constructed NSUN2^−/−^ and NSUN6^−/−^ HEK293T cell lines and performed relevant biological analysis. The deletion of NSUN2 and NSUN6 resulted in diminished cell proliferation and increased expression of the senescence-associated marker P27. Additionally, cells with methyltransferase deletions exhibited a higher number of β-galactosidase-positive cells in response to oxidative stress induced by H_2_O_2_. Furthermore, we observed a significant reduction in NSUN2 and NSUN6 expression in the HGPS senescent cell model generated by the LMNA^G609G^ mutation. This together suggest that NSUN2 and NSUN6 may be inextricably linked to aging, providing new avenues for combating aging and treating aging-related diseases.

## Data Availability

The original contributions presented in the study are included in the article/Supplementary Material, further inquiries can be directed to the corresponding author.
