# Supplementary figures and images for: Unveiling the potential impact of RNA m5C methyltransferases NSUN2 and NSUN6 on cellular aging
Source: Front Genet. 2025 Apr 16;16:1477542. doi: 10.3389/fgene.2025.1477542 (PMC12040966; doi:10.3389/fgene.2025.1477542)

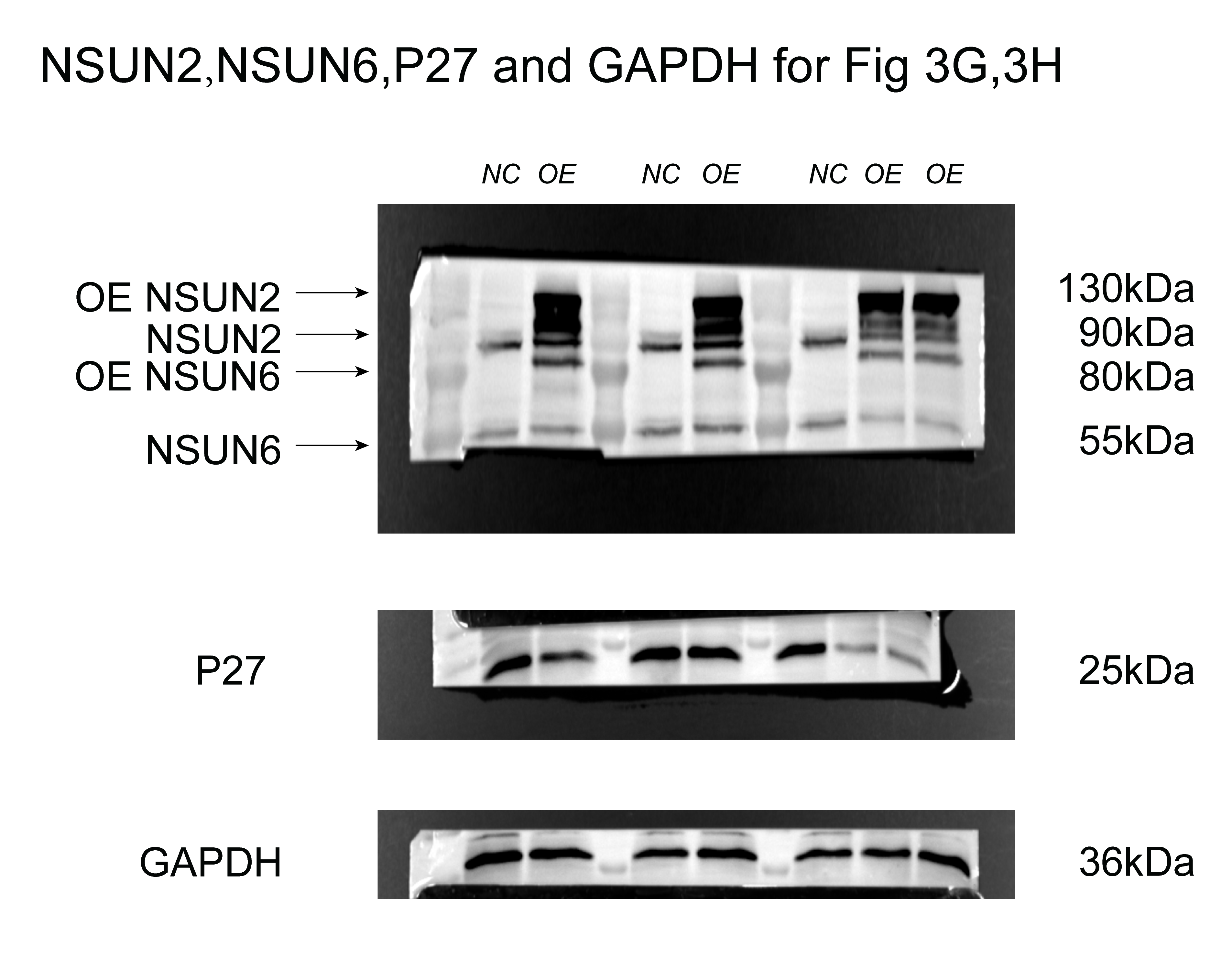

Supplement: Supplementary file 1 [file Image6.tif]

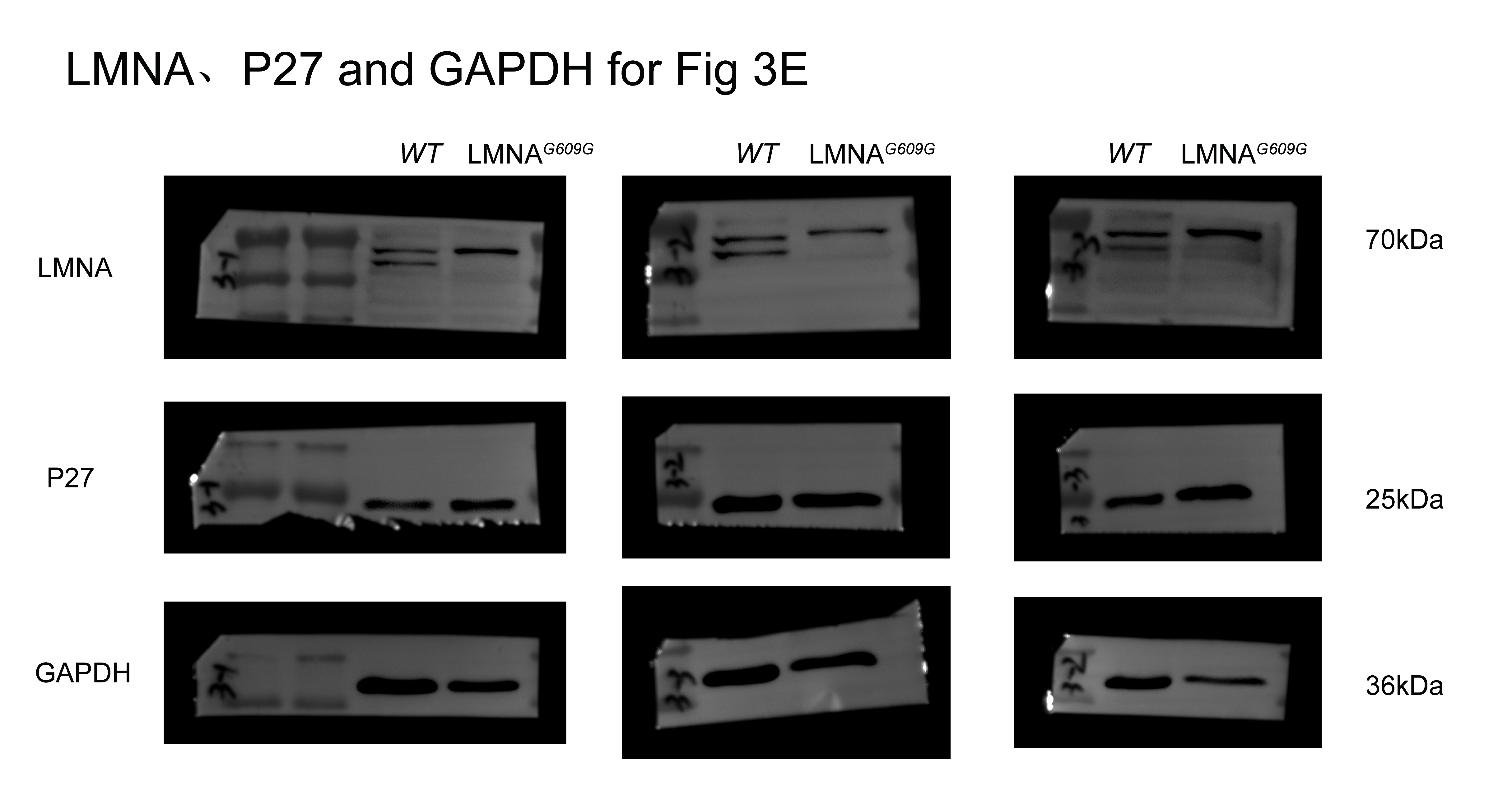

Supplement: Supplementary file 3 [file Image3.tif]

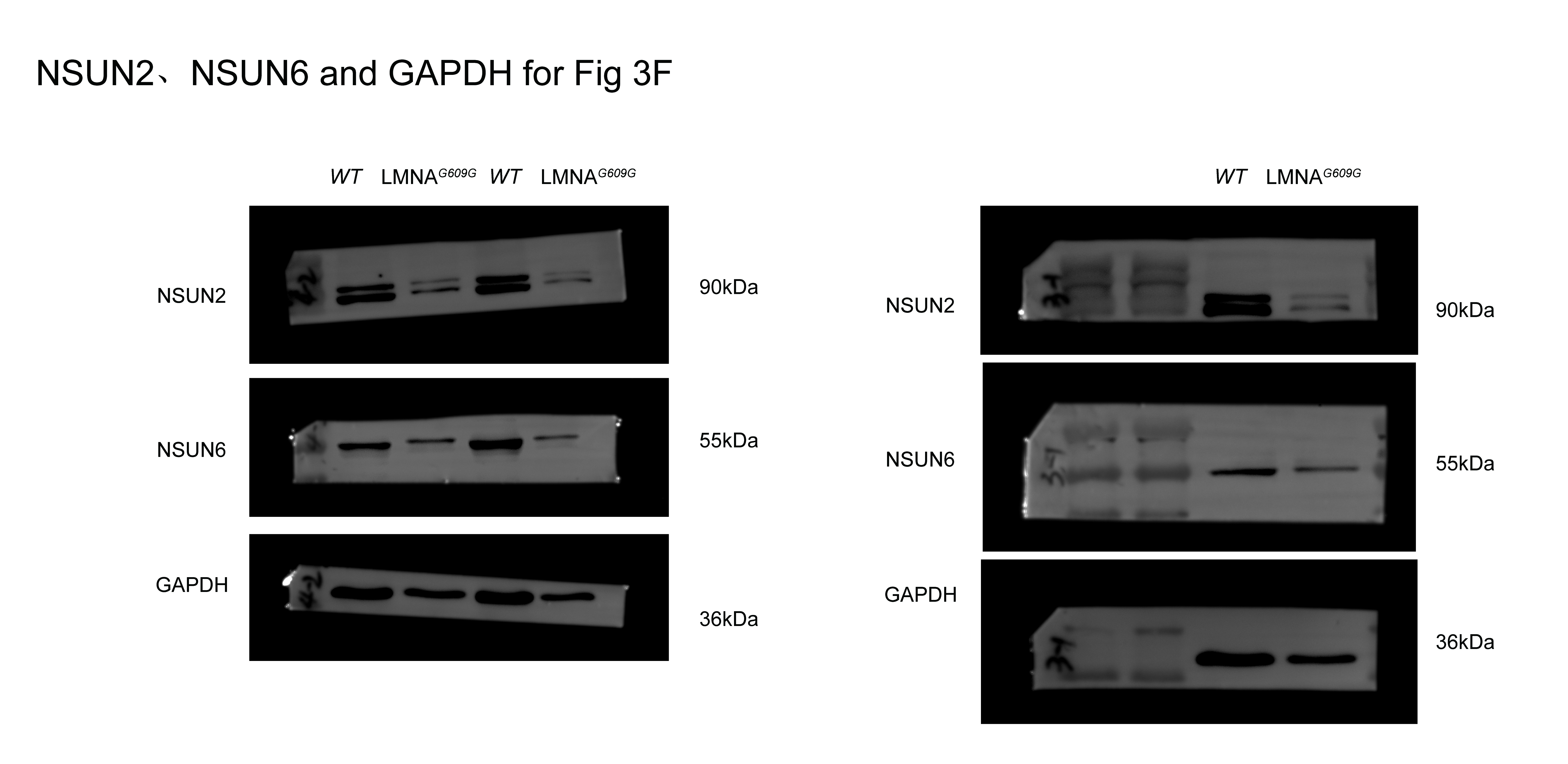

Supplement: Supplementary file 4 [file Image4.tif]

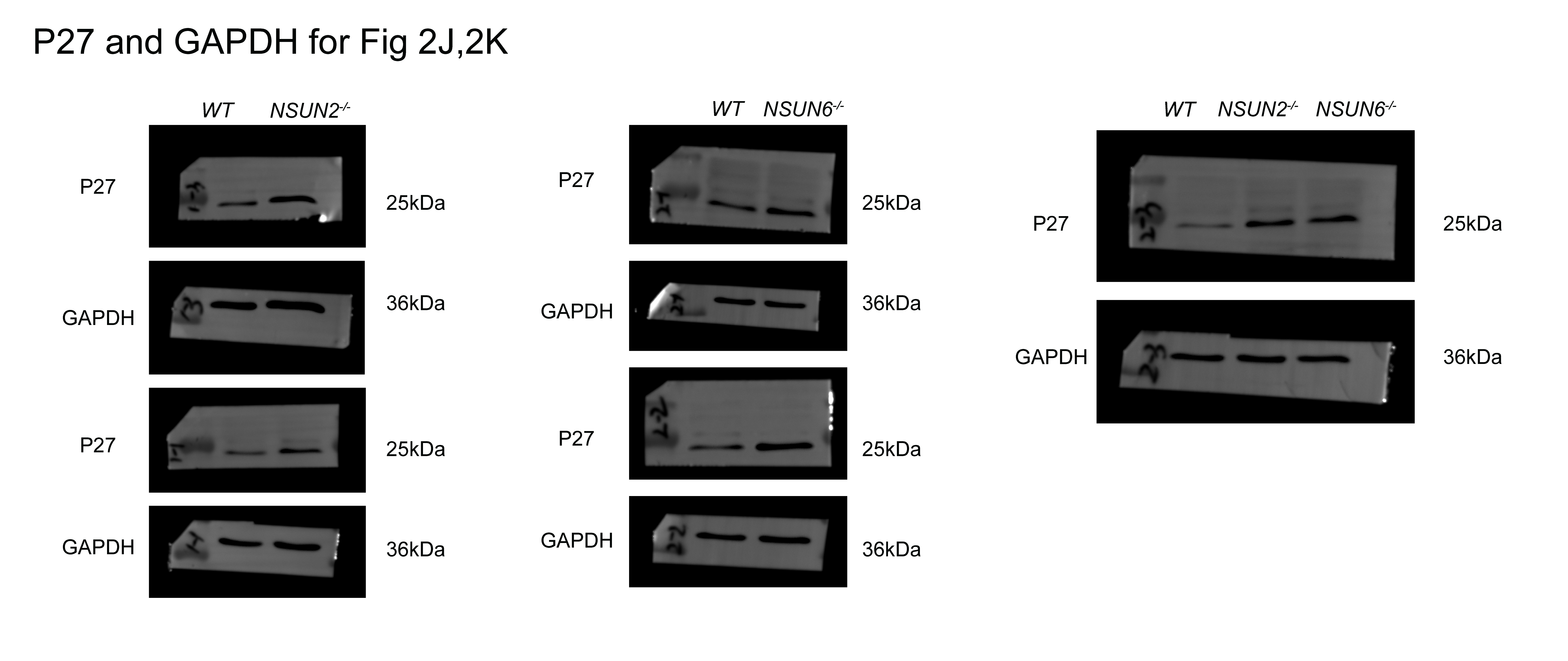

Supplement: Supplementary file 5 [file Image2.tif]

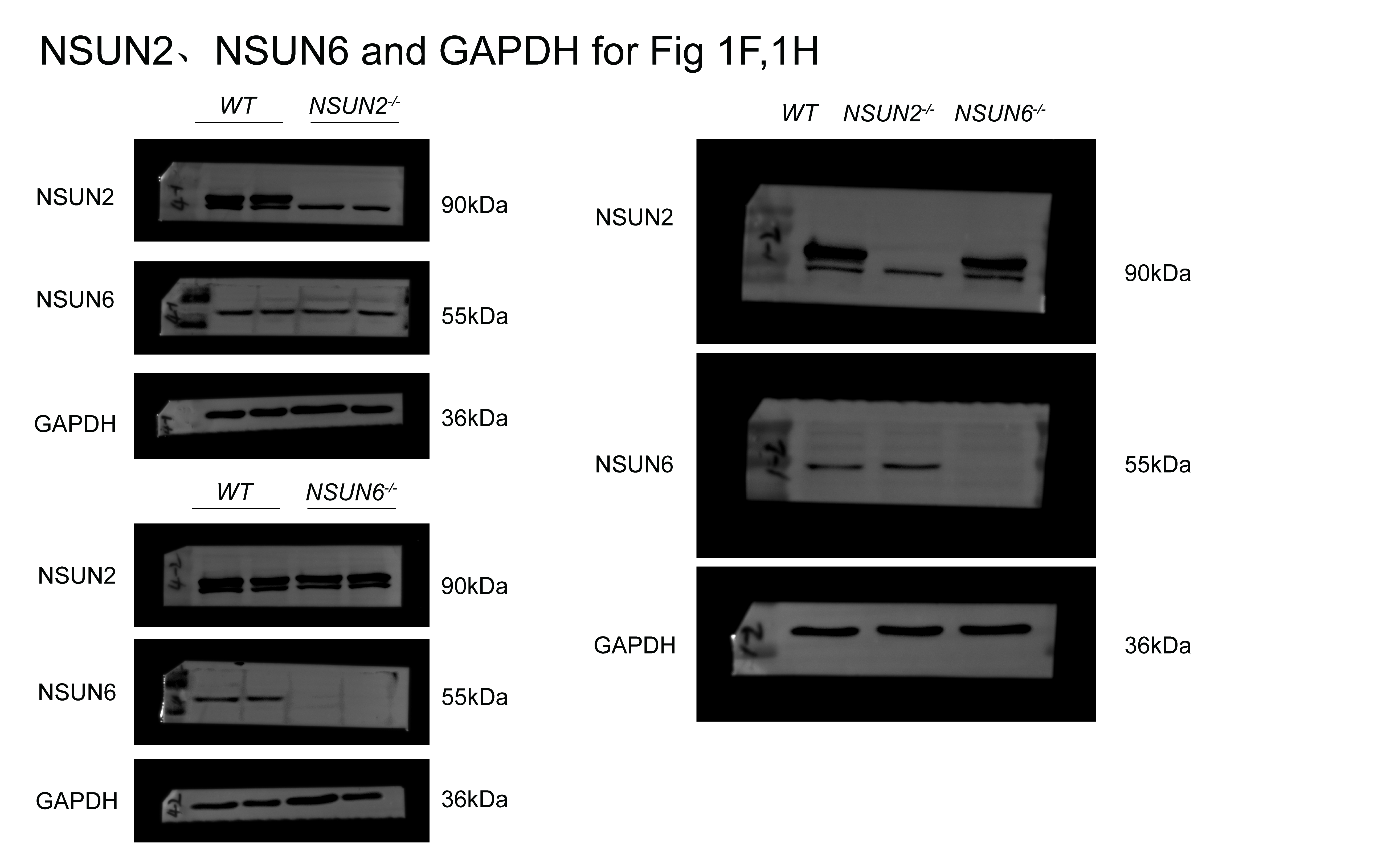

Supplement: Supplementary file 6 [file Image1.tif]

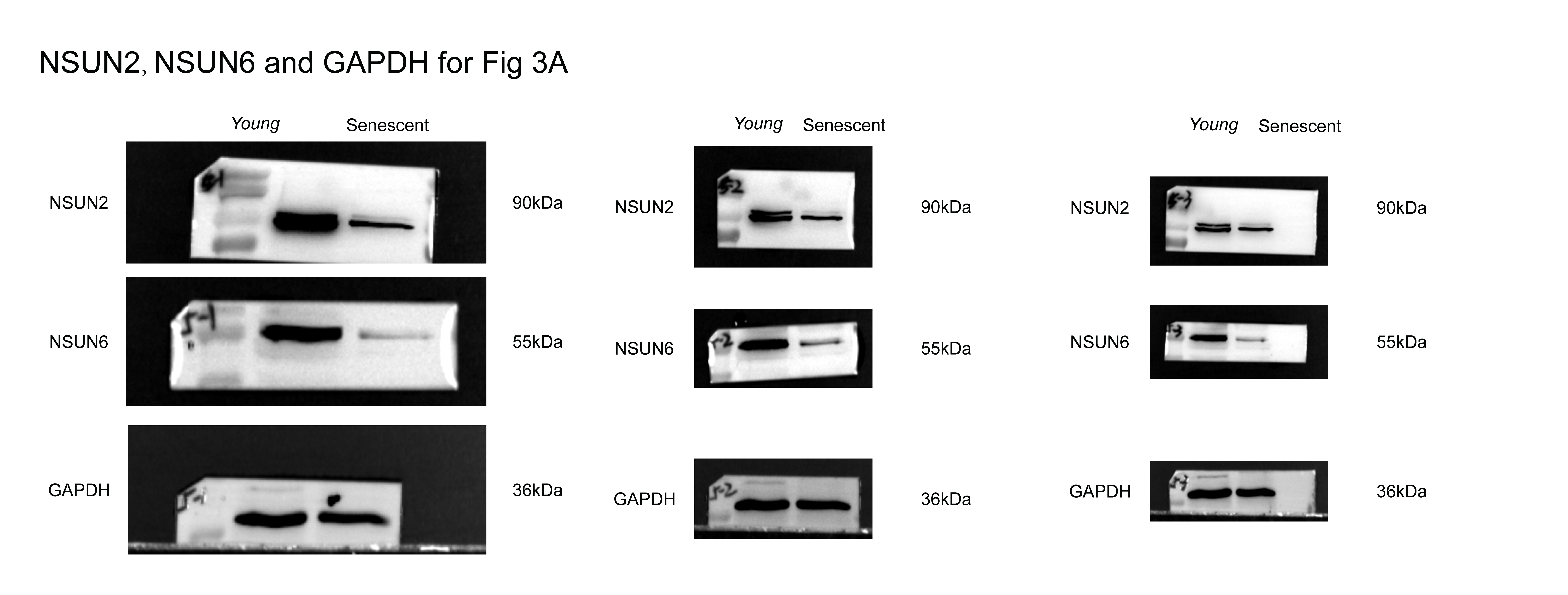

Supplement: Supplementary file 7 [file Image5.tif]
