# Supplementary material for: Unveiling the potential impact of RNA m5C methyltransferases NSUN2 and NSUN6 on cellular aging
Source: Front Genet. 2025 Apr 16;16:1477542. doi: 10.3389/fgene.2025.1477542 (PMC12040966; doi:10.3389/fgene.2025.1477542)
Supplement: Supplementary file 2 [file Table1.docx]

**Supplementary Table 1. The primers used for genotyping and single guide RNA**

| **Name of Primer** | **Sequences (5′−3′)** |
| --- | --- |
| NSUN2-KO -PCR-Fwd | CCCCTTAGAGCTGTTCGCTGT |
| NSUN2-KO -PCR-Rev | GTCGAAGAAAACACCGTCGCCTT |
| NSUN6-KO -PCR-Fwd | TACCATGTTGAAGCCCAAGAA |
| NSUN6-KO -PCR-Rev | GAAGCTACTAAGGCCCAGTTT |
| NSUN2-PX459sgRNA1-H-Fwd | CACCGCGCCATCCTCCGCGTCCTC |
| NSUN2-PX459sgRNA1-H-Rev | AAACGAGGACGCGGAGGATGGCGC |
| NSUN2-PX459sgRNA2-H-Fwd | CACCGGGTGGTGGAAAGCGCGGCG |
| NSUN2-PX459sgRNA2-H-Rev  NSUN2-PX459sgRNA3-H-Fwd  NSUN2-PX459sgRNA3-H-Rev  NSUN2-PX459sgRNA4-H-Fwd  NSUN2-PX459sgRNA4-H-Rev  NSUN6-PX459sgRNA1-H-Fwd  NSUN6-PX459sgRNA1-H-Rev  NSUN6-PX459sgRNA2-H-Fwd  NSUN6-PX459sgRNA2-H-Rev  LMNA-BE3sgRNA-H-Fwd  LMNA-BE3sgRNA-H- Rev  LMNA -PCR-Fwd  LMNA -PCR- Rev | AAACCGCCGCGCTTTCCACCACCC  CACCGAGGCTACCCCGAGATCGTCA  AAACTGACGATCTCGGGGTAGCCTC  CACCGTGTTCTCCTTGACGATCTCG  AAACCGAGATCGTCAAGGAGAACAC  CACCTAGGTAAACAAGAAGCAGAA  AAACTTCTGCTTCTTGTTTACCTA  CACCATTTTTCACATGTTGTACTG  AAACCAGTACAACATGTGAAAAAT  CCGGGTGGGCGGATCCATCTCCTC  AAACGAGGAGATGGATCCGCCCAC  GTGCCGGTCCTAAGAAGTCA GTCTCCCCAACCCTCACACT |
